# Positive and negative effects of collaboration on suggestibility and false memory in online groups

**DOI:** 10.1007/s12144-023-04775-y

**Published:** 2023-05-24

**Authors:** Clelia Rossi-Arnaud, Pietro Spataro, Alessandro Santirocchi, Maria Chiara Pesola, Laura Costantini, Vincenzo Cestari

**Affiliations:** 1 Department of Psychology, Sapienza University of Rome, Via dei Marsi 78, 00185 Rome, Italy; 2 Faculty of Economics, Universitas Mercatorum, Piazza Mattei 10, Rome, 00186 Italy

**Keywords:** Computer-mediated communication, Collaborative inhibition, Suggestibility, False memory

## Abstract

Previous studies demonstrated the positive and negative effects of collaboration on memory (both veridical and false recall) and suggestibility in face-to-face contexts. However, it remains unclear whether the same results can be observed in a virtual context. To clarify this issue, the present study examined the performance of 10 nominal triads and 10 collaborative triads in a fully online setting. Participants interacted live, in videoconference and were tested with the Gudjonsson Suggestibility Scale (GSS) and the Deese/Roediger-McDermott (DRM) task. For the GSS, the results replicated the in-person pattern of results, with collaborative triads showing the standard inhibition effect in the immediate and delayed (after 24 h) recall tasks; in addition, collaborative triads were less suggestible than nominal triads. For the DRM, we likewise found that collaboration decreased the recall and recognition of both studied items (the standard inhibitory effect) and critical lures (the error-pruning effect). We therefore conclude that remembering in a virtual context exhibits the same general properties as its in-person counterpart, at least when using a videoconference setting.

## Positive and negative effects of collaboration on veridical memory, false memory, and suggestibility in electronic groups

From a historical perspective, most studies investigating memory processes have been conducted on single individuals working alone. More recently, however, researchers have shown an interest in investigating how memories might be socially formed (for reviews see Harris et al., 2008; Maswood & Rajaram, 2019; Rajaram & Pereira-Pasarin, 2010; Rajaram, 2011). Typically, these studies compared the memory performance of collaborative groups (in which participants are asked to collaborate to recall, or recognize, as much information as possible) with that of individual groups (in which participants are asked to work alone, without interacting in any way). Several interesting findings have emerged from this body of research, highlighting both the negative and the positive effects of collaboration on veridical and false memory.

### Negative and positive effects of collaboration on veridical and false memory

First, the classical studies by Basden et al. (1997) and Weldon and Bellinger (1997) showed that, although collaborative groups recall more correct items than the mean of individual groups, the collaborative group performance is usually less accurate than that of nominal groups. The latter is determined by summing the outputs of individuals working alone but counting redundant items only once (Basden et al., 1997; Weldon & Bellinger, 1997). The dominant explanation for this *collaborative inhibition* effect is the so-called *retrieval disruption hypothesis*, according to which every individual uses an idiosyncratic strategy to retrieve the studied items, which would be disrupted by exposure to the recall outputs of the other members (Basden et al., 1997; Finlay et al., 2000; Wright & Klumpp, 2004; see Marion & Thorley 2016, for a meta-analysis). In addition to the disruption of retrieval strategies, other cognitive mechanisms might also be involved in producing the memory deficit of collaborative groups. For example, retrieval inhibition refers to the idea that the items produced by each member can act as part-set cues, by impairing the recall of non-retrieved items by other participants (Barber et al., 2015). On the other hand, there is little evidence to support the role of production blocking, evaluation apprehension and social loafing mechanisms (Hinds & Payne, 2016; Weldon et al., 2000).

Second, previous studies have demonstrated that collaborative groups are less likely to produce false items in tests of free recall or to incorrectly recognize non-studied lures in tests of recognition (Henkel & Rajaram, 2011; Ross et al., 2004, 2008; Rossi-Arnaud et al., 2011; Takahashi, 2007; Vredeveldt et al., 2016). The *error-pruning* hypothesis account for these results by assuming that the members of collaborative groups can cross-check the quality of each other’s responses and use corrective feedbacks to discard memory errors (Rajaram & Pereira-Pasarin, 2010; Rajaram, 2011). A similar prediction follows from the *grounding framework*, which looks at the processes by which collaborating participants seek and provide evidence to establish mutual understanding. This account assumes that collaborators seek to minimize the efforts required to reach consensus. It predicts that, if members can see and hear each other as well as easily produce speaking turns and nonverbal cues, they should rely primarily on group filtering processes to remove incorrect items from the collective product (Clark & Wilkes-Gibbs, 1986; Ekeocha & Brennan, 2008; Ross et al., 2004, 2008; Takahashi, 2007) suggested instead that collaboration might inhibit the production of wrong answers. If collaborating individuals are uncertain about the accuracy of their memories, they may choose to accept their partners’ memories instead of providing their own.

The positive impact of collaboration on the recall of false memories is particularly evident when using the Deese/Roediger-McDermott paradigm (DRM: Deese 1959; Roediger & McDermott, 1995), in which participants are asked to study and immediately recall a series of semantically related lists – i.e., lists composed of words conceptually related to non-studied critical lures. At the end of the presentation of all lists, they are given a recognition test that includes studied words and critical lures (Roediger & McDermott, 1995). Most of the studies examining the effects of collaboration in the DRM task used however a modified procedure in which participants were first presented with all the semantically related lists and then performed a single recall task, either individually or collaboratively (i.e., the final recognition task was not administered). Overall, these experiments reached consistent conclusions, by showing that nominal groups recalled more studied words and more critical lures than collaborative groups (Maki et al., 2008; Maswood et al., 2022; Nie et al., 2022; Saraiva et al., 2017; Weigold et al., 2014; but see Thorley & Dewhurst 2009, for different conclusions): thus, collaboration inhibited the recall of correct (studied) items but simultaneously reduced the recall of incorrect (lure) items.

### Effects of collaboration on suggestibility

The research briefly reviewed above has provided important evidence regarding the positive and negative effects of collaboration on veridical and false memory. A series of studies have recently expanded these results by investigating the impact of collaboration on interrogative suggestibility (Rossi-Arnaud et al., 2019, 2021; see also Rossi-Arnaud et al., 2020) – here defined as “the extent to which, within a closed social interaction, people come to accept messages communicated during formal questioning, as a result of which their subsequent behavioral response is affected” (Gudjonsson & Clark, 1986). This was achieved by using the Gudjonsson Suggestibility Scale (Gudjonsson, 1984, 1987), an instrument in which participants hear a story, provide immediate and delayed recall, and finally answer a set of misleading questions (both before and after receiving negative feedback about their performance). The GSS has the advantage of measuring both traditional memory performance and interrogative suggestibility. In their first study, Rossi-Arnaud et al. (2019) administered the GSS2 (involving a non-forensic story) to 10 nominal dyads and 16 collaborative dyads. The important result was that, compared to nominal pairs, collaborative pairs produced less confabulated elements in the free recall tasks and were considerably less prone to give in to misleading questions, both before and after the negative feedback. Thus, collaboration had a beneficial impact on suggestibility, by reducing the participants’ willingness to incorrectly accept misleading questions.

These findings were later confirmed by Rossi-Arnaud et al. (2021), who also investigated whether group-filtering contributed to the lower suggestibility of collaborative triads. To measure these processes, Rossi-Arnaud et al. (2021) computed the so-called inclusive scores (Harris et al., 2012) – i.e., scores that included the cases in which at least one participant in the collaborative triad accepted the misleading question but was later corrected by other group members during discussion (such that the affirmative responses were not included in the final output of the group). When this was done, it turned out that the difference between nominal and collaborative groups in the tendency to endorse misleading questions was eliminated, at least prior to receiving the negative feedback (the advantage of collaborative groups was likewise reduced after the negative feedback but remained significant).

### Aims of the present study and predictions

Considering this background, the primary aim of our study was to determine whether the positive and negative effects of collaboration in the GSS and DRM tasks, typically observed in face-to-face groups, could be replicated in an online setting. As stated by Greeley et al. (2022), the study of memory in virtual environments is still in its infancy, leaving many questions unanswered. For the purposes of the present study, the crucial issue was whether social remembering in a virtual context exhibited the same general properties as its in-person counterpart. The few studies investigating this issue reached mixed conclusions (Ekeocha & Brennan, 2008; Hinds & Payne, 2016, 2018). Greeley et al. (2022) employed a fully online, chat-based environment and reported that collaborative inhibition was eliminated in these conditions, because nominal groups recalled less than their counterparts in face-to-face conditions. In this respect, it is worth noting that our online setting differed from that illustrated by Greeley et al. (2022), since participants did not interact via a chat box: rather, they met in the Zoom platform and performed the memory tasks live, in videoconference. Compared to chat-based platforms, interactions through Zoom or Google Meet are more similar to social exchanges taking place in face-to-face since group members have access to both verbal and non-verbal information (i.e., gestures, emotional expressions, and so on). Previous studies support the idea that synchronous communication, which requires all participants to be ‘present’ at the same time, generates greater collaborative efforts among distant learners and enhances socio-emotional aspects of collaborative learning processes, as compared to asynchronous communication (which is often based on time-delayed interactions; Chou 2002; Serçe et al., 2011). Thus, studying whether individuals are prone to accept misleading information communicated during social online questioning represents a theoretically relevant question, which has not been previously assessed.

A second, subsidiary aim was to determine whether the negative and positive effects of collaboration persisted after a delay of 24 h. Previous research has begun to investigate delayed suggestibility by using a modified paradigm in which participants took the immediate recall task and answered the GSS questions in the first session and were then administered the delayed recall task on a separate session, after two days (Wachi et al., 2019) or after a one-week delay (Gudjonsson et al., 2016; Vagni et al., 2015). This procedure allowed a measurement of delayed suggestibility, indicated by the number of misleading suggestions provided during Yield 1 and Yield 2 which had become incorporated into the participant’s delayed recall of the story. Since we were not specifically interested in examining the effects of collaboration on delayed suggestibility, we simply manipulated the delay between the immediate and delayed recalls. To this purpose, we asked participants (a) to hear the story and provide an immediate recall in the first session, and then (b) to provide a delayed recall and answer the GSS questions (two times, before and after receiving the negative feedback) in the second session, which occurred after a 24-hour delay - rather than after the standard 50-minute delay recommended by the GSS instructions.

Our predictions were as follows. Regarding collaborative inhibition, the retrieval disruption hypothesis (Basden et al., 1997; Weldon & Bellinger, 1997; Wright & Klumpp, 2004) suggests that, regardless of interactional condition (face-to-face vs. virtual environment), nominal groups should retrieve more correct story elements than collaborative groups in the GSS 1 immediate and delayed recall tasks. They should also recall and recognize more correct words in the DRM task. This is because in both face-to-face and virtual online conditions, each participant is exposed to the recall products of the other group members, which should interfere with the use of individual retrieval strategies. In other words, the retrieval disruption hypothesis predicts that collaborative inhibition should be significant in our online setting (and similar in size to that obtained in face-to-face conditions). On the other hand, the recent study by Greeley et al. (2022) leads to a different expectation. As mentioned above, these authors found that online, nominal participants were less motivated to complete the task, which eliminated the advantage over collaborative groups typically obtained in face-to-face conditions. According to the *reverse social loafing* hypothesis, participants working individually in a remote online condition may feel less responsible for group output and less incentivized to recall more than a handful of items (Greeley et al., 2022). In contrast, participants working in group may experience increased engagement and motivation, due to the social component involved in their condition. A substantial decrease in performance might occur in our nominal participants, leading to the elimination of collaborative inhibition. Hinds and Payne (2018) made a similar prediction, claiming that computer-mediated communication may allow group members to partially disregard others’ contributions and utilize personal retrieval strategies more efficiently than in face-to-face situations.

Regarding the positive effects of collaboration, both the error-pruning hypothesis (Rajaram & Pereira-Pasarin, 2010; Rajaram, 2011) and the grounding framework (Clark & Wilkes-Gibbs, 1986; Ekeocha & Brennan, 2008) predict that participants working in a collaborative condition where they can see and hear each other (as in our setting) should rely primarily on other group members to filter incorrect memories from their joint outputs. Previous studies have shown that group filtering helps to reduce memory errors and suggestibility in collaborative groups (Harris et al., 2012; Rossi-Arnaud et al., 2021). Given the high similarity of our online condition to face-to-face interactions, we predicted that collaborative groups would be less suggestible than nominal groups in the GSS1 and would recall and recognize less critical lures in the DRM task. The inhibition hypothesis put forward by Ross et al. (2004, 2008) and Takahashi (2007) leads to the same expectation, although the underlying mechanism should be quite different. Specifically, members of collaborative groups should adopt a more conservative response criterion than members of nominal groups, making them less likely to accept misleading questions and contribute their memories in recall and recognition tasks.

## Method

### Participants

A total of 60 students at the University [masked for peer review] volunteered to participate in the present experiment. They were randomly assigned to 10 nominal triads and 10 collaborative triads. Nominal triads were composed of 20 females and 10 males (age: *M* = 21.63 years, *SD* = 1.96; education: *M* = 15.00 years, *SD* = 1.84), while collaborative triads were composed of 19 females and 11 males (age: *M* = 23.57 years, *SD* = 1.81; education: *M* = 16.13 years, *SD* = 1.80). Care was taken to ensure that participants working in the same triad were unfamiliar to each other, since previous studies suggested that collaborative inhibition may be reduced in groups of friends (Andersson, 2001; Andersson & Rönnberg, 1995).

## Materials

### Gudjonsson Suggestibility Scale (GSS)

The Gudjonsson Suggestibility Scale (Gudjonsson, 1984, 1987; Bianco & Curci, 2015) was used to investigate individual differences in memory performance and interrogative suggestibility. Two parallel versions of the instrument are available, which differ only in the content of the story (forensic for the GSS 1, non-forensic for the GSS 2). The GSS 1, which was used in the present study, comprises a short story describing a robbery, divided in 40 items (these items are clearly identified and separated by slashes in the scoring sheet), plus a set of 20 questions: five of them asked about true events that were really mentioned in the story (control questions), while the other fifteen questions included incorrect details that were never presented in the original story (misleading questions; e.g., “Was the name of the woman Anna Balducci?” when the correct name was Anna Colucci).

### Deese/Roediger-McDermott (DRM) task

The Deese/Roediger–McDermott paradigm (DRM: Deese, 1959; Roediger & McDermott, 1995; Italian version by: Iacullo & Marucci, 2016) was used to study the production of false memories. Materials included (a) 12 lists of 15 words each, that were semantically related to a non-presented critical lure (e.g., the word ‘Sweet’ was the critical lure for the list including: Candy, Amaro, Pastry, Tender, Sugar, Honey, Greedy, Tart, Acrid, Flavour, Cake, Cream, Whipped Cream, Fruit, Salted) and (b) a 72-item, paper-based, yes–no recognition test containing 36 list items (three from each of the 12 presented lists), 12 critical lures from the presented lists, and 24 nonlist items (i.e., novel items that were never presented to participants). The nonlist items were unrelated to both lists items and critical lures and were selected from the CoLFIS vocabulary (https://www.istc.cnr.it/en/grouppage/colfiseng). Note that we selected the twelve lists that were more likely to lead to false memories, based on the data reported by Iacullo and Marucci (2016). We did so because we wanted to maximize the possibility to observe the error-pruning effects of collaboration.

### Procedure

Since the present experiment was run during the COVID-19 pandemic (from 21 to 2020 to 26 August 2021), all the sessions took place on the Zoom platform, in line with the Italian government’s recommendations on limiting face-to-face interactions. A link was initially shared by the experimenter with the selected participants (via email). When all of them were connected, the experimenter sent an informed consent to each participant, with the instruction to fill in the form and send it back to the experimenter. After having collected all the informed consents, the procedure began.

**Fig. 1:**
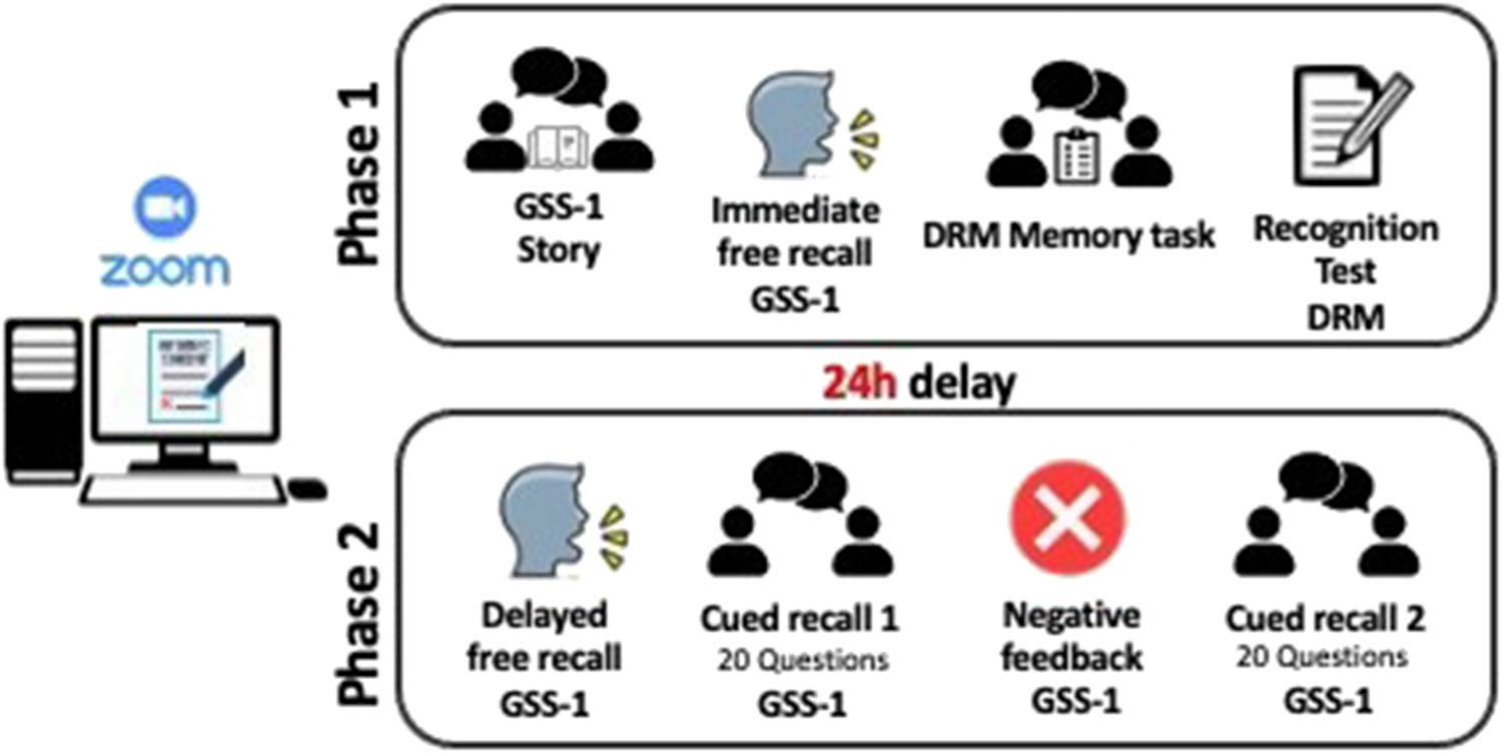
A schematic illustration of the experimental procedure

Each session involved two phases, separated by a 24-hour delay (see Fig. 1). During the first phase, the experimenter read out the GSS 1 story to the participants, who were instructed to listen carefully (“I would like you to listen to a short story. Please listen carefully because, when I will have finished, I want you to recall all the information you remember”). At the end of the story, they were asked to provide an immediate free recall account (“Now please recall everything you remember from the story”). At this point, the session continued with the administration of the DRM task. Following Iacullo and Marucci (2016), the experimenter presented the twelve lists to the participants, with the instruction to memorize the words in view of an immediate recall task. Each word was presented at the center of the experimenter’s screen (which was shared with all participants) for 2 s (using 44-point font). At the end of each list, participants were instructed to write down all the words that they could remember. This procedure was repeated for all the lists. After the final recall test, participants were administered the paper-based recognition test and asked to indicate on the appropriate response sheet which words were presented in the previous twelve lists.

The second session occurred after a 24-hour delay. The same participants who took part in the first phase were sent a second link via email and the experimenter checked that all of them were connected before proceeding. At this point, the participants were asked to provide a delayed free recall of the GSS 1 story (the instructions were the same as those used in the immediate test). At the end of the delayed recall, they had to answer the set of 20 questions included in the GSS 1 a first time (“I’m going to ask you some questions about the story. Try to be as accurate as possible”). When the participants had responded to all the questions, the experimenter left the session for some minutes by stating that he/she had to check the participants’ performance (the videocamera was darkened during this period but, unbeknownst to participants, the experimenter remained connected online). At his return, the experimenter provided a negative feedback and asked participants to answer the 20 questions a second time (“You have made a number of errors. It is therefore necessary to go through the questions once more, and this time try to be more accurate”).

Participants included in the nominal triads worked alone throughout the entire session, without collaborating. Each of them was previously sent all the response sheets (prepared using Google Form) via email and the experimenter checked that they did not interact with each other during the administration of the tasks. In contrast, participants included in the collaborative triads were allowed to interact. Specifically, they were told to actively collaborate during the memory tasks to remember as many details (in the GSS 1 immediate and delayed recall tasks) and words (in the DRM free recall and recognition tasks) as possible; in the case of the GSS 1 questionnaire, they were instructed to reach the consensus before giving a common response to each question (Rossi-Arnaud et al., 2019). One participant was chosen at the beginning of the session to type the responses produced by the group and was therefore sent all the response sheets. Following Weldon and Bellinger (1997), participants working in the collaborative triads were given no specific instructions on how to resolve potential disagreements (free-for-all procedure). The present research was approved by the Ethical Committee of the University Sapienza of Rome (Protocol N.00002199) and was performed in accordance with the ethical standards as laid down in the 1964 Declaration of Helsinki and its later amendments.

### Measures and data coding

The GSS 1 allowed us to assess the following measures: (a) *Immediate recall*: the number of correct items recalled immediately after hearing the story (range 0–40); (b) *Immediate confabulation*: the number of fabricated (new) or distorted (modified) items reported in the immediate recall; (c) *Delayed recall*: the number of correct items recalled after the 24-hour delay (range 0–40); (d) *Delayed confabulation*: the number of items fabricated or distorted in the delayed recall; (e) *Yield 1*: the number of misleading questions to which participants gave in when responding to the questionnaire, before the administration of the negative feedback (range 0–15); (f) *Yield 2*: the number of misleading questions to which participants gave in after the administration of the negative feedback (range 0–15); (g) *Shift*: the number of times participants changed their answers to the control and misleading questions after receiving the negative feedback (range 0–20); (h) *Total Suggestibility*: the sum of Yield 1 and Shift scores, which reflects participants’ overall suggestibility (range 0–35).

The GSS manual provides detailed information on how to compute the immediate and delayed free recall scores (see Rossi-Arnaud et al., 2019, for examples). Specifically, 1 point in the free-recall tasks was assigned whenever the general concept underlying a given item was clearly reported, even if the exact wording was different from that used in the story. When the item comprised two different elements (e.g., “Anna Colucci” included both the name and the surname of the protagonist), a score of 0.5 point was assigned whenever the participants retrieved only one correct element (only “Anna” or only “Colucci”). For the immediate and delayed confabulation scores, we coded both distorted and fabricated items. A distorted detail occurred when the original item underwent substantial changes without altering the overall meaning, whereas a fabricated detail referred to the case in which participants reported an element that was never presented in the story. Both instances were assigned 1 point.

For the questionnaire, responses to the leading questions such as “Yes”, “I think yes”, “It’s possible”, “Maybe” were considered as yield responses and were assigned 1 point. In contrast, responses such as “I don’t remember”, “This information was not mentioned in the story”, “I don’t know”, “I’m not sure” were not considered as yield responses and were therefore assigned 0 points. Lastly, as concerns the shift scores, the manual stipulates that the changes in the responses provided before and after the negative feedback had to be clear-cut to be taken as positive evidence. For example, changes from “no” to “yes”, from “don’t know” to “yes”, or from “don’t remember” to “yes” were considered as substantial changes and were therefore assigned 1 point. In contrast, changes from “don’t remember” to “no”, from “maybe” to “no”, or from “it’s possible” to “yes” were not sufficient to be regarded as response shifts and were therefore assigned 0 points.

Following an established tradition in this field (e.g., Basden et al., 1997; Weldon & Bellinger, 1997), the scores obtained by the three participants working in each nominal triad were pooled together, with redundant items counted only once^1^. For example, for the immediate and delayed free recall task, an item was considered redundant if at least two participants were assigned 1 point. That is, both participants had to remember the general concept conveyed by a given item, even if they expressed it in different wordings. A special case occurred when two (or more) participants were assigned 0.5 points to the same item. In this condition, the recall was considered redundant (and assigned 0.5 points) if the participants retrieved the same portion of the compound item (e.g., if both participants recalled that the name of the protagonist was “Anna”). Otherwise, if the participants reported different portions of the same item (e.g., one member reported only “Anna” and the other member reported only “Colucci”), their individual scores were pooled to 1 point (0.5 + 0.5). The same procedure as above was followed for the GSS 1 questionnaire. Hence, individual scores were pooled together, by considering “redundant” the questions for which two (or more) participants provided an affirmative response. For example, suppose that one participant responded “yes” to the misleading questions 3, 7, 11, 15, whereas another participant responded affirmatively to the misleading questions 3, 7, 10, 19, 20: in this case, their pooled score was seven (i.e., the sum of the points assigned to the questions 3, 7, 10, 11, 15, 19, and 20).

For the DRM task, the following measures were obtained from the recall phase: (a) *List recall*: the proportions of items correctly recalled for each list (range: 0–15), and (b) *Lure recall*: the proportions of lure items incorrectly recalled across the 12 lists (range: 0–12). For the final recognition task, three measures were assessed: (c) *Correct hit rates*: the proportions of list items correctly recognized (range: 0–36); (d) *critical lure FAR (false alarm rates)*: the proportions of lure items incorrectly recognized (range: 0–12); (e) *unrelated FAR*: the proportions of non-list items incorrectly recognized (range: 0–24). For each measure, nominal scores were again computed by pooling individual responses and counting redundant items only once.

## Results

### GSS 1 task

**Fig. 2:**
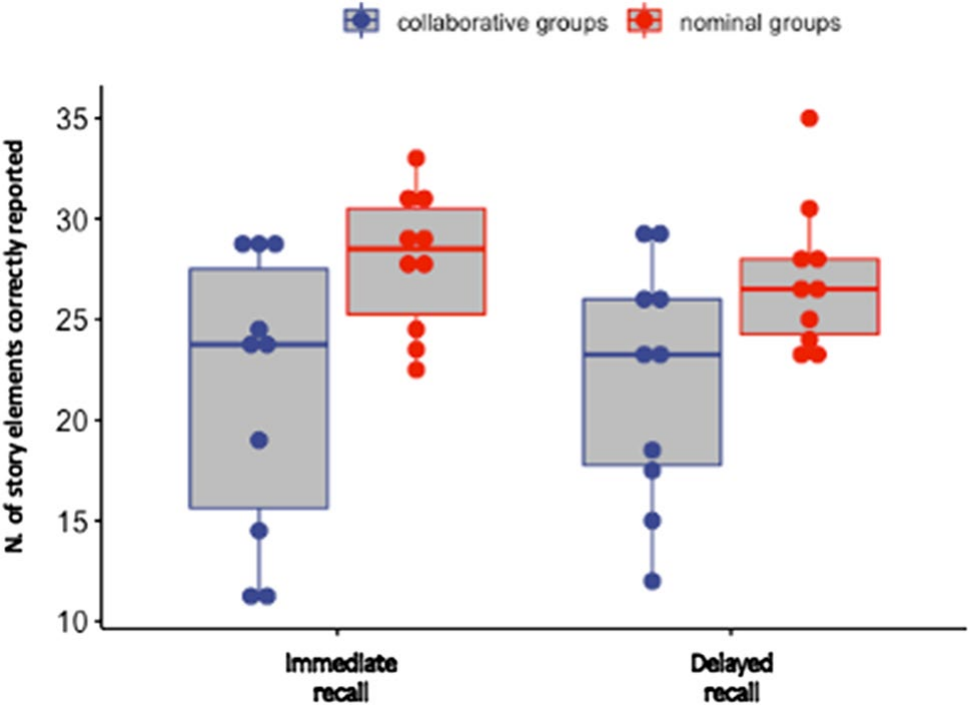
Boxplots illustrating the number of story elements correctly recalled by nominal and collaborative groups in the immediate and delayed recall tasks. The solid lines within each box plot represent median values

Immediate and delayed free recall scores were analyzed with a mixed ANOVA, considering Group (nominal vs. collaborative) as a between-subjects factor and Time (immediate vs. delayed recall) as a within-subjects factor. As illustrated in Fig. 2, the results revealed a significant main effect of Group [*F*(1, 18) = 6.40, MSE = 55.61, *p* = .021, *η*_p_^2^ = 0.26], indicating that nominal triads (*M* = 27.45) recalled more correct story items than collaborative triads (*M* = 21.70). Thus, a significant collaborative inhibition effect was observed in our online setting. The main effect of Time and the interaction between Group and Time were not significant [*F*(1, 18) = 0.06, MSE = 3.27, *p* = .79, *η*_p_^2^ < 0.01 and *F*(1, 18) = 1.71, MSE = 3.27, *p* = .20, *η*_p_^2^ = 0.09, respectively]^2^.

**Fig. 3:**
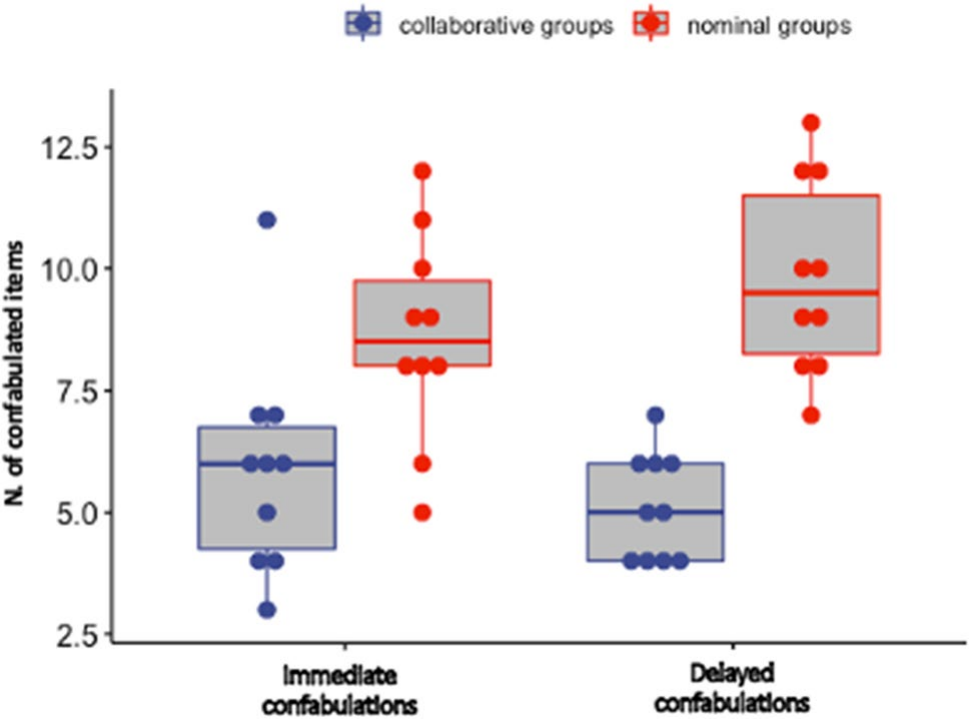
Boxplots illustrating the number of confabulations produced by nominal and collaborative groups in the immediate and delayed recall tasks. The solid lines within each box plot represent median values

The same mixed ANOVA as above was applied to the immediate and delayed confabulation scores, as well as to the yield scores obtained by nominal and collaborative groups. For confabulation scores (see Fig. 3), the results showed a main effect of Group [*F*(1, 18) = 24.84, MSE = 5.51, *p* < .001, *η*_p_^2^ = 0.58], indicating that nominal triads (*M* = 9.20) produced more confabulated items than collaborative triads (*M* = 5.50). This finding confirms the error-pruning effects of collaboration previously reported by Rossi-Arnaud et al. (2019). The main effect of Time was not significant [*F*(1, 18) = 0.22, MSE = 1.81, *p* = .64, *η*_p_^2^ = 0.01], but the interaction between Group and Time reached the significance level [*F*(1, 18) = 5.52, MSE = 1.81, *p* = .030, *η*_p_^2^ = 0.24]. A follow-up analysis of simple effects revealed that the difference between nominal and collaborative groups was significant in both the immediate and delayed recall phases [*F*(1, 18) = 7.69, MSE = 4.73, *p* = .013, *η*_p_^2^ = 0.29 and *F*(1, 18) = 42.75, MSE = 2.58, *p* < .001, *η*_p_^2^ = 0.70, respectively]. On the other hand, the effect of Time was marginal for nominal triads [*F*(1, 18) = 3.97, *p* = .062, *η*_p_^2^ = 0.18], indicating a slight increase in the production of confabulations in the delayed recall phase (from *M* = 8.60 to *M* = 9.80), but non-significant for collaborative triads [*F*(1, 18) = 1.76, *p* = .20, *η*_p_^2^ = 0.09].

**Fig. 4:**
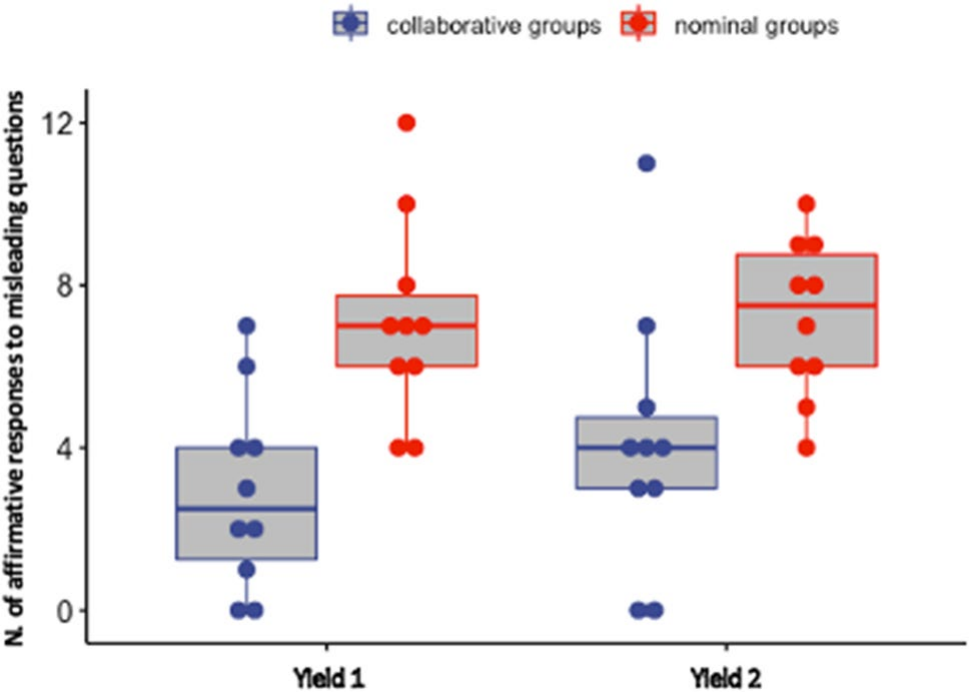
Boxplots illustrating the yield scores obtained by nominal and collaborative groups. The solid lines within each box plot represent median values

For yield scores (see Fig. 4), the results showed only a significant main effect of Group [*F*(1, 18) = 12.75, MSE = 10.44, *p* = .002, *η*_p_^2^ = 0.41], indicating that nominal triads (*M* = 7.15) produced more affirmative responses to misleading questions than collaborative triads (*M* = 3.50). Thus, collaborative groups were less likely to give up to misleading questions, again confirming the conclusions reached by Rossi-Arnaud et al. (2019). The main effect of Time and the interaction between Group and Time were not significant [*F*(1, 18) = 1.71, MSE = 2.45, *p* = .20, *η*_p_^2^ = 0.09 and *F*(1, 18) = 1.23, MSE = 2.45, *p* = .18, *η*_p_^2^ = 0.06, respectively].

Lastly, the shift scores and the total suggestibility scores were analyzed with two *t*-tests for independent samples, considering Group (nominal vs. collaborative) as the between-subjects factor. For shift scores, no significant results were obtained [*t*(18) = 0.47, *p* = .64, Cohen’s *d* = 0.21]. In contrast, the total suggestibility scores were significantly higher for nominal (*M* = 11.10) than for collaborative scores (*M* = 6.40) [*t*(18) = 2.30, *p* = .034, Cohen’s *d* = 1.03].

### DRM task

The mean list and lure recall proportions were analyzed with a *t*-test for independent samples, considering Group (nominal vs. collaborative) as the between-subjects factor. Significant results were obtained in both cases. Specifically, list recall was significantly higher for nominal (*M* = 0.86) than for collaborative (*M* = 0.77) groups [*t*(18) = 3.33, *p* = .004, Cohen’s *d* = 1.49], again reflecting the standard inhibitory effect of collaboration. Lure recall was also higher for nominal (*M* = 0.56) than for collaborative (*M* = 0.22) groups [*t*(18) = 5.53, *p* < .001, Cohen’s *d* = 2.47], confirming that collaboration reduced the recall of non-presented words.

For the recognition task, we likewise performed a series *t*-test for independent samples, considering Group (nominal vs. collaborative) as the between-subjects factor. The results showed that both the correct hit rates and the critical lure FARs were higher for nominal than for collaborative groups [for correct hit rates: *M* = 0.97 vs. *M* = 0.91, *t*(18) = 3.98, *p* = .001, Cohen’s *d* = 2.00; for critical lure FARs: *M* = 0.94 vs. *M* = 0.48, *t*(18) = 5.41, *p* < .001, Cohen’s *d* = 2.53]. In other words, collaboration inhibited the recognition of studied words, but simultaneously reduced the recognition of lure words. Interestingly, a significant difference was also observed on the unrelated FARs, since nominal groups incorrectly recognized more non-list words than collaborative groups [*M* = 0.17 vs. *M* = 0.06, *t*(18) = 4.87, *p* < .001, Cohen’s *d* = 2.42].

We previously noted that the collaborative reduction in the recognition of false lures in the DRM could be explained by proposing that participants refrained from providing their own recollections because they were uncertain about the accuracy of their memories (Ross et al., 2004, 2008; Takahashi, 2007). In the context of a standard yes/no recognition task, this hypothesis implies that participants working in collaborative groups should adopt a more conservative criterion, as compared to those working in nominal groups. We tested this idea in the DRM by computing the sensitivity (*d*ʹ) and bias (*C*) measures suggested by the Signal Detection Theory (Macmillan & Creelman, 1991). Briefly, sensitivity represents the observer’s ability to respond in accordance with the true nature of the presented stimulus (i.e., to respond “yes” to studied stimuli and “no” to unstudied stimuli), while response bias is the observer’s tendency to use one of the two responses. Negative values of *C* indicate a liberal bias toward responding yes, whereas positive values indicate a conservative bias toward the no response. To adjust for cases in which either the hit rate was equal to 1 or the FAR was equal to 0, we used the recommendations illustrated by Stanislaw and Todorov (1999, p.144). The obtained data were again analyzed with two *t*-test for independent samples, considering Group (nominal vs. collaborative) as the between-subjects factor. The results showed that nominal and collaborative groups did not differ in terms of sensitivity [*M* = 2.86 vs. *M* = 2.94, *t*(18) = − 0.62, *p* = .54, Cohen’s *d* = 0.30]. However, collaborative groups were significantly more conservative than nominal groups [*M* = 0.07 vs. *M* = − 0.50, *t*(18) = − 4.49, *p* < .01, Cohen’s *d* = 2.03], thus confirming the conclusions previously reached by Ross et al. (2004, 2008) and Takahashi (2007).

A similar procedure was applied to critical lure FARs. In this case the results indicated that sensitivity was lower for collaborative than for nominal groups [*M* = 1.48 vs. *M* = 2.46, *t*(18) = 3.11, *p* = .006, Cohen’s *d* = 1.40]; in addition, collaborative groups were more conservative than nominal groups [*M* = 0.66 vs. *M* = − 0.29, *t*(18) = − 5.64, *p* < .001, Cohen’s *d* = 2.54]. Thus, collaborative groups were less likely to falsely recognize critical lures and were more conservative in the false recognition of unrelated lures.

### Inclusive scores

As explained in the Introduction, one way to verify the role of group-filtering processes in the reduction of collaborative groups’ suggestibility is to look at the discussions occurring in these groups and compute the so-called inclusive scores (Harris et al., 2012). For yield 1 and yield 2 measures, we therefore added to the original scores the cases in which at least one member of the collaborative group gave in to a misleading question but was later corrected by other members. These scores were again analyzed with a mixed ANOVA, considering Group (nominal vs. collaborative) as a between-subjects factor and Time (before vs. after the negative feedback) as a within-subjects factor. The results showed that the main effect of Group did not reach the significance level [*F*(1, 18) = 3.24, MSE = 11.12, *p* = .088, *η*_p_^2^ = 0.15], although yield scores were still numerically higher for nominal triads (*M* = 7.15) than for collaborative triads (*M* = 5.25). The main effect of Time and the interaction between Group and Time were not significant [*F*(1, 18) = 1.62, MSE = 2.21, *p* = .21, *η*_p_^2^ = 0.08 and *F*(1, 18) = 1.12, MSE = 2.21, *p* = .30, *η*_p_^2^ = 0.05, respectively].

Similarly, for the DRM, we added to the lure recall scores and the critical lure FARs the cases in which at least one member of the collaborative group falsely recognized a lure word as a studied item but was later corrected by other members during the discussion. These scores were again analyzed with a *t*-test for independent samples, considering Group (nominal vs. collaborative) as the between-subjects factor. Here, the results showed that both the lure recall scores and the critical lure FARs continued to be higher for nominal than for collaborative groups [for lure recall: *M* = 6.75 vs. *M* = 3.65, *t*(18) = 3.48, *p* = .003, Cohen’s *d* = 1.55; for critical lure FARs: *M* = 11.37 vs. *M* = 7.57, *t*(18) = 2.78, *p* = .012, Cohen’s *d* = 1.24]. In sum, the use of group-filtering processes accounted for the collaborative reduction in yield scores (in the GSS) but did not fully eliminate the collaborative advantage in the rejection of lure items (in the DRM).

## Discussion

The present study investigated the positive and negative effects of collaboration in the GSS and DRM tasks by using a completely online setting. Our primary aim was to determine whether virtual social remembering produced the same effects as those obtained in face-to-face social remembering (Greeley et al., 2022). The results can be summarized as follows.

### Positive and negative effects of collaboration in the GSS

Replicating previous findings (Rossi-Arnaud et al., 2019, 2021), our analyses indicated that collaborative groups recalled significantly less correct elements than nominal groups in the immediate and delayed recall tasks of the GSS1. Thus, a standard collaborative inhibition effect was obtained in our virtual setting. This finding aligns well with the predictions following from the retrieval disruption hypothesis (Basden et al., 1997; Weldon & Bellinger, 1997). In both face-to-face and online collaborative conditions, each participant hears the recall products of the other group members, causing a significant disruption in the use of individual retrieval strategies.

On the other hand, our results are inconsistent with those reported by Greeley et al. (2022, Exp.1). Using a fully online chat-based environment, these authors reported that collaborative inhibition was eliminated in a word recall task, primarily because participants of nominal online groups performed worse than participants of nominal face-to-face groups (no difference was observed between online and face-to-face collaborative groups). To account for their data, Greeley et al. (2022) proposed the *reverse social loafing hypothesis*, according to which nominal participants working online would feel less responsible for the group output and less incentivized to provide their best performance (Greeley et al., 2022). A follow-up experiment showed that the standard collaborative inhibition effect could be restored when the presence of the experimenter was made clear and the instructions provided participants with information about the typical performance of ‘other participants’, to reduce social loafing and evaluation apprehension (Greeley et al., 2022, Exp.2).

To evaluate whether a reverse social loafing effect was at play in our study, we tried to determine whether memory performance of our online participants was also lower than that observed in face-to-face conditions. Due to the ongoing pandemic, we did not include an in-person condition in the present study (and thus, we could not draw a direct comparison); we referred to a previous experiment assessing GSS 1 performance in face-to-face nominal and collaborative triads (Rossi-Arnaud et al., 2021). The comparison was limited to immediate recall and confabulation because the interval before the delayed recall task was different in the two experiments (Rossi-Arnaud et al. adopted a 50-minute interval, while the present study used a 24-hour delay). Briefly, we found that face-to-face triads recalled significantly more correct details than virtual triads in the immediate recall; however, unlike Greeley et al. (2022), the disadvantage of the virtual condition applied to both nominal and collaborative triads. For immediate confabulation, virtual triads reported significantly more incorrect details than face-to-face triads. As in the previous analysis, the difference occurred for both nominal and collaborative groups^3^. Hence, the present data extend those reported by Greeley et al. (2022), by showing that participants working in virtual environments produced less accurate memories, irrespectively of whether they were assigned to nominal or collaborative triads. One possible explanation is that working online allows for more distractions and mind-wandering than working in a controlled laboratory setting (Marsh & Rajaram, 2019; Pereira-Pasarin & Rajaram, 2011). The use of a remote environment might therefore lead to a shallower encoding of the GSS1 story.

In terms of the positive effects of collaboration, our findings are consistent with the predictions derived from the grounding framework (Clark & Wilkes-Gibbs, 1986; Ekeocha & Brennan, 2008). This account suggests that when members of collaborative groups can see and hear each other and easily produce both verbal and nonverbal cues (as is the case in the current online environment), they should rely primarily on other group members to filter out incorrect memories from the collective product. Each participant cross-checks the quality and accuracy of the collaborators’ output and provides corrective feedbacks when necessary. The efficacy of group filtering processes in reducing false memories was demonstrated in studies dealing with episodic memory (Harris et al., 2012; Ross et al., 2004, 2008; Takahashi, 2007). Furthermore, in the study by Rossi-Arnaud et al. (2021), the difference in suggestibility between nominal and collaborative groups was eliminated when the scoring included cases in which at least one collaborative group participant accepted a misleading question, but the response was corrected by other group members and thus did not enter the final output. The present analyses replicated this finding and showed that it holds true even when misleading questions are presented 24 h after encoding (rather than after 50 min, as in Rossi-Arnaud et al., 2021). This is a relevant finding, because, in real forensic contexts, people are often questioned about witnessed events after long delays. It was therefore important to determine whether collaborative remembering could reduce suggestibility over long-term periods.

The grounding framework also predicts that collaborative participants interacting via a chat box should rely primarily on self-filtering processes to assess the suitability of their recollections for inclusion in the group output (Ekeocha & Brennan, 2008). This is due to the significantly higher costs of producing a turn and/or receiving a response in the written modality, making it more efficient for individuals to self-filter their contributions. The implication is that, if the positive effects of collaboration on suggestibility are primarily driven by group-filtering processes occurring in live contexts, then the probability to observe them should be lower when using asynchronous means of communication. Collaborative inhibition should be likewise reduced in the latter condition, because participants interacting via chat box are less likely to pay attention to their collaborators’ contributions and thus are less negatively affected by the typical disruption of retrieval strategies that occurs in face-to-face conditions (Hinds & Payne, 2016, 2018). Although the present study cannot evaluate these predictions, understanding the way in which different forms of virtual communication moderate the positive and negative effects of collaboration on memory and suggestibility represents, in our opinion, an interesting avenue for future research.

### Positive and negative effects of collaboration in the DRM

Regarding the DRM, our results confirmed the coexistence of the positive and negative effects of collaboration previously reported, among the others, by Maki et al. (2008), Maswood et al. (2022), Saraiva et al. (2017) and Weigold et al. (2014). In fact, the analyses showed that nominal groups recalled more list (studied) words than collaborative groups – again replicating the standard collaborative inhibition effect; however, a similar increase was observed for the recall of critical lures, suggesting that the beneficial effects of collaboration generalized to the DRM paradigm.

The same pattern occurred in the recognition task: both the correct hit rates and the critical lure FARs were higher for nominal than for collaborative groups. Interestingly, however, when we computed sensitivity (*d*ʹ) scores, it turned out that the difference between nominal and collaborative groups was no longer significant. Thus, nominal and collaborative participants did not differ in their ability to discriminate between studied and unstudied words. The elimination of the collaborative inhibition effect in a recognition task is consistent with the predictions following from the retrieval disruption hypothesis (see Barber et al., 2010, 2015; Finlay et al., 2000). In a recall task, each participant retrieves the studied stimuli according to the order dictated by an idiosyncratic retrieval strategy, making his/her performance open to the disruption caused by the exposure to the recall products of the other group members (Basden et al., 1997; Weldon & Bellinger, 1997). In contrast, in a recognition task, the studied stimuli are provided in a fixed order, which is the same for all participants. Since this format does not rely on the use of one’s own organizational strategies, the expected output is that collaborative inhibition should be reduced or eliminated – a pattern confirmed by the present study.

Turning to the positive effects of collaboration, our data are in contrast with previous findings indicating that collaboration can facilitate (rather than reduce) the recognition of critical lures (Thorley & Dewhurst, 2009) explained this outcome by speculating that, during the discussion, the members of collaborative groups might be more likely to vividly remember a critical lure being studied and to offer convincing, but incorrect, recall-based arguments to support their judgements. Our results are at odds with this hypothesis and instead suggest that collaboration in the DRM paradigm leads to the opposite outcome – a reduction in the recognition of critical lures. Interestingly, it appears that this benefit was produced by mechanisms that were, at least in part, different from those explaining the collaborative reduction in suggestibility. This is because the follow-up analyses involving inclusive scores revealed that, while the advantage of collaborative groups in the recognition of critical lures was numerically reduced, it was still significant. Hence, the advantage could not be exclusively attributed to the use of group-filtering processes.

As stated in the Introduction, an alternative possibility, previously advanced by Ross et al. (2008) and Takahashi (2007), is that collaboration might induce a conservative change in the response criterion adopted by participants. That is, the members of collaborative groups might be less likely to give ‘old’ responses, irrespective of the nature of the items (studied or unstudied). In line with this proposal, the analysis of bias measures indicated that collaborative groups were significantly more conservative than nominal groups during the DRM recognition task. Clearly, when people interact, their behaviors are not only driven by task-related needs, but also by social needs (Ekeocha & Brennan, 2008). One crucial point in this respect is face management – i.e., the necessity to preserve one’s own self-esteem. If one group member contributes an idea he/she is uncertain about and the other members recall it correctly, the risk is to lose credibility. These considerations might lead participants to produce only those memories for which they have high levels of certainty.

### Conclusions and limitations

To conclude, the present study demonstrated that collaborative remembering had both negative and positive effects in the GSS and DRM paradigms, and that these effects persisted in a virtual setting where participants met via videoconference and performed the memory tasks live. The negative effects were demonstrated by the collaborative inhibition in the GSS’s immediate and delayed recall of story elements, as well as in the DRM’s recall of studied words. Collaborative inhibition was instead eliminated in the DRM recognition task, at least when the analyses were focused on *d*ʹ scores. Positive effects, on the other hand, were indicated by a lower tendency of collaborative groups to give in to misleading questions (in the DRM) and a reduction in the proportions of critical lures incorrectly recalled or recognized as old (in the GSS). In the GSS, the positive effects of collaboration were primarily explained by the use of group-filtering processes; in contrast, in the DRM recognition, it appears that collaboration induced participants to adopt a more conservative response criterion.

Clearly, these results must be evaluated with caution, since, as stated above, we included neither a face-to-face condition nor a virtual condition in which participants’ interactions occurred through chat messages. The inclusion of a face-to-face condition is essential to verify whether the use of a virtual environment leads to a shallower encoding of the study materials. Likewise, the inclusion of a chat-based condition may be necessary to determine whether participants working in these conditions pay less attention to others’ contributions and rely primarily on self-filtering processes (Ekeocha & Brennan, 2008). Future studies will need to manipulate the way in which collaborative groups interact in real and virtual environments, in order to assess the positive and negative effects of collaborative remembering in a wide range of conditions.

## Data Availability

Data will be provided upon request to the corresponding author.

## Appendix 1

In the present Appendix 1 we report the results of the analyses performed by comparing individual and collaborative groups. For individual groups, scores were computed by averaging the individual performance of each participant (see Rossi-Arnaud et al., 2011).

### GSS 1 task

Immediate and delayed free recall scores were analyzed with a mixed ANOVA, considering Group (individual vs. collaborative) as a between-subjects factor and Time (immediate vs. delayed recall) as a within-subjects factor. The results revealed a significant main effect of Group [*F*(1, 18) = 8.81, MSE = 44.68, *p* = .008, *η*_p_^2^ = 0.33], indicating that collaborative triads (*M* = 21.70) recalled more correct story items than individual triads (*M* = 15.42). Thus, the standard collaborative facilitation effect was observed in our online setting. The main effect of Time and the interaction between Group and Time were not significant [*F*(1, 18) = 0.09, MSE = 3.79, *p* = .75, *η*_p_^2^ < 0.01 and *F*(1, 18) = 1.65, MSE = 3.79, *p* = .21, *η*_p_^2^ = 0.08, respectively].

The same mixed ANOVA as above was applied to the immediate and delayed confabulation scores, as well as to the yield scores obtained by individual and collaborative groups. For confabulation scores, the results showed a main effect of Group [*F*(1, 18) = 8.31, MSE = 2.86, *p* = .010, *η*_p_^2^ = 0.31], indicating that collaborative triads (*M* = 5.50) produced more confabulated items than individual triads (*M* = 3.95). The main effect of Time was not significant [*F*(1, 18) = 0.02, MSE = 1.23, *p* = .87, *η*_p_^2^ < 0.01], but the interaction between Group and Time reached the significance level [*F*(1, 18) = 4.46, MSE = 1.23, *p* = .049, *η*_p_^2^ = 0.19]. A follow-up analysis of simple effects revealed that the difference between collaborative and individual groups was significant in the immediate recall phases [*F*(1, 18) = 8.68, MSE = 3.00, *p* = .009, *η*_p_^2^ = 0.33], but not in the delayed recall phase [*F*(1, 18) = 2.92, MSE = 1.09, *p* = .10, *η*_p_^2^ = 0.14].

For yield scores, the results showed only a marginal main effect of Time [*F*(1, 18) = 3.96, MSE = 1.54, *p* = .062, *η*_p_^2^ = 0.18], indicating that the proportions of affirmative responses to misleading questions increased slightly after the negative feedback (*M* = 3.08 vs. *M* = 3.86). The main effect of Group and the interaction between Group and Time were not significant [*F*(1, 18) = 0.003, MSE = 7.76, *p* = .95, *η*_p_^2^ < 0.01 and *F*(1, 18) = 1.12, MSE = 1.54, *p* = .30, *η*_p_^2^ = 0.05, respectively].

### DRM task

The mean list and lure recall proportions were analyzed with a *t*-test for independent samples, considering Group (individual vs. collaborative) as the between-subjects factor. Significant results were obtained only for list recall, which was significantly higher for collaborative (*M* = 0.77) than for individual (*M* = 0.56) groups [*t*(18) = − 7.63, *p* < .001, Cohen’s *d* = 3.68], again reflecting the standard effect of collaborative facilitation. Lure recall did not differ between collaborative (*M* = 0.22) and individual (*M* = 0.24) groups [*t*(18) = 0.32, *p* = .74, Cohen’s *d* = 0.17], confirming that collaboration reduced the recall of non-presented words.

For the recognition task, we likewise performed a series *t*-test for independent samples, considering Group (individual vs. collaborative) as the between-subjects factor. The results showed that correct hit rates were higher for collaborative than for individual groups [*M* = 0.91 vs. *M* = 0.72, *t*(18) = − 6.11, *p* < .001, Cohen’s *d* = 3.14]. Critical lure FARs were marginally higher for individual (*M* = 0.64) than for collaborative (*M* = 0.48) groups [*t*(18) = 1.82, *p* = .085, Cohen’s *d* = 0.86], whereas no differences were detected for unrelated FARs [*M* = 0.06 vs. *M* = 0.08, *t*(18) = 0.83, *p* = .41, Cohen’s *d* = 0.52].

## Footnotes

1 The same analyses were repeated by averaging the scores obtained by each participant of the nominal groups. The results were comparable to those illustrated in previous studies (e.g., Rossi-Arnaud et al., 2011; Weldon & Bellinger, 1997) and are reported in the Appendix 1.

2 The absence of the main effect of Time is surprising, because the present study adopted a relatively long delay between the immediate and delayed recalls. A potential explanation might reside in the structured nature of to-be-remembered material. Brief stories, such as those presented in the GSS1, allow participants to use schemas to support their recall and might therefore minimize time-related interference effects (Marion & Thorley, 2016).

3 For immediate recall, a 2 (Condition: face-to-face vs. virtual) × 2 (Group: nominal vs. collaborative) between-subjects ANOVA found significant main effects of both factors [*F*(1, 41) = 15.46, MSE = 24.55, *p* < .001, *η*_p_^2^ = 0.27 for Condition, and *F*(1, 41) = 13.43, MSE = 24.55, *p* = .001, *η*_p_^2^ = 0.24 for Group]. Face-to-face triads (*M* = 30.55) recalled more correct details than virtual triads (*M* = 24.65), and nominal triads (*M* = 30.35) recalled more correct details than collaborative triads (*M* = 24.85). The two-way interaction was not significant [*F*(1, 41) = 0.44, MSE = 24.55, *p* = .51, *η*_p_^2^ = 0.01]. Similarly, when applied to immediate confabulation scores, the same ANOVA as above showed significant main effects of both Condition [*F*(1, 41) = 45.55, MSE = 3.48, *p* < .001, *η*_p_^2^ = 0.52] and Group [*F*(1, 41) = 26.60, MSE = 3.48, *p* < .001, *η*_p_^2^ = 0.39]. Face-to-face triads (*M* = 3.43) recalled less incorrect details than virtual triads (*M* = 7.25), and collaborative triads (*M* = 3.88) recalled less incorrect details than collaborative triads (*M* = 6.80). The two-way interaction did not reach the significance level [*F*(1, 41) = 0.14, MSE = 24.55, *p* = .70, *η*_p_^2^ = 0.00].
